# The Impact of the Latest Danian Event on Planktic Foraminiferal Faunas at ODP Site 1210 (Shatsky Rise, Pacific Ocean)

**DOI:** 10.1371/journal.pone.0141644

**Published:** 2015-11-25

**Authors:** Sofie Jehle, André Bornemann, Arne Deprez, Robert P. Speijer

**Affiliations:** 1 Institut für Geophysik und Geologie, Universität Leipzig, Talstr. 35, 04103 Leipzig, Germany; 2 Bundesanstalt für Geowissenschaften und Rohstoffe, Stilleweg 2, 30655 Hannover, Germany; 3 Department of Earth and Environmental Sciences, KU Leuven, Celestijnenlaan 200E, 3001 Leuven/Heverlee, Belgium; Ben Gurion University of the Negev, ISRAEL

## Abstract

The marine ecosystem has been severely disturbed by several transient paleoenvironmental events (<200 kyr duration) during the early Paleogene, of which the Paleocene-Eocene Thermal Maximum (PETM, ~56 Ma) was the most prominent. Over the last decade a number of similar events of Paleocene and Eocene age have been discovered. However, relatively little attention has been paid to pre-PETM events, such as the “Latest Danian Event” ("LDE", ~62.18 Ma), specifically from an open ocean perspective. Here we present new foraminiferal isotope (δ^13^C, δ^18^O) and faunal data from Ocean Drilling Program (ODP) Site 1210 at Shatsky Rise (Pacific Ocean) in order to reconstruct the prevailing paleoceanographic conditions. The studied five-meter-thick succession covers ~900 kyr and includes the 200-kyr-lasting LDE. All groups surface dwelling, subsurface dwelling and benthic foraminifera show a negative δ^13^C excursion of >0.6‰, similar in magnitude to the one previously reported from neighboring Site 1209 for benthic foraminifera. δ^18^O-inferred warming by 1.6 to 2.8°C (0.4–0.7‰ δ^18^O measured on benthic and planktic foraminiferal tests) of the entire water column accompanies the negative δ^13^C excursion. A well stratified upper ocean directly before and during the LDE is proposed based on the stable isotope gradients between surface and subsurface dwellers. The gradient is less well developed, but still enhanced after the event. Isotope data are supplemented by comprehensive planktic foraminiferal faunal analyses revealing a dominance of *Morozovella* species together with *Parasubbotina* species. Subsurface-dwelling *Parasubbotina* shows high abundances during the LDE tracing changes in the strength of the isotope gradients and, thus, may indicate optimal living conditions within a well stratified surface ocean for this taxon. In addition, distinct faunal changes are reported like the disappearance of *Praemurica* species right at the base of the LDE and the continuous replacement of *M*. *praeangulata* with *M*. *angulata* across the LDE.

## Introduction

Over the past two decades, a series of short-term warming events (<200 kyr) have been documented for the early Palaeogene (c. 66–48 Ma, e.g. [1–3]). Many of these events have in common that they are associated with abrupt perturbations of the global carbon cycle as reflected in the δ^13^C of inorganic and biogenic carbonates as well as of terrestrial and marine organic matter, an accompanying temperature rise and extreme biotic responses. The most prominent of these events is the Paleocene-Eocene Thermal Maximum (PETM; 56 Ma; e.g., [4, 5]. This event is characterized by (a) a short-lived 170–230 kyr lasting, negative δ^13^C excursion (CIE) between 1 and 5‰ (e.g., [6, 7]), (b) global warming of several degrees as indicated by temperature-sensitive proxies like δ^18^O, Mg/Ca or TEX_86_ [5], (c) a drop of CaCO_3_ in deep-sea sediments and a shallowing of the lysocline/CCD probably due to ocean acidification [8], and (d) a major extinction of deep-sea benthic foraminifera and changes in organic dinoflagellate, larger foraminifera, ostracoda and calcareous plankton communities (e.g. [9–13]). The strong negative CIE indicates that the amount of isotopically light carbon added to the global carbon cycle, possibly derived from methane hydrates, is to some extent comparable to the present day input of carbon to the atmosphere through combustion of fossil fuels. Thus, the PETM is sometimes considered as a deep-time analogue to rapid climate change as is expected for the near future (e.g. [2, 14, 15]).

Recent studies have revealed that the PETM may not have been a single event. Similar events of Paleocene age are the Dan-C2 Event (65.2 Ma ago) [16], the Latest Danian Event (LDE) or Top Chron C27n Event (62.18 Ma, e.g. [17–20]), and the Mid- Paleocene Biotic Event (MPBE, 58.9 Ma, e.g. [21, 22].

**Fig 1:**
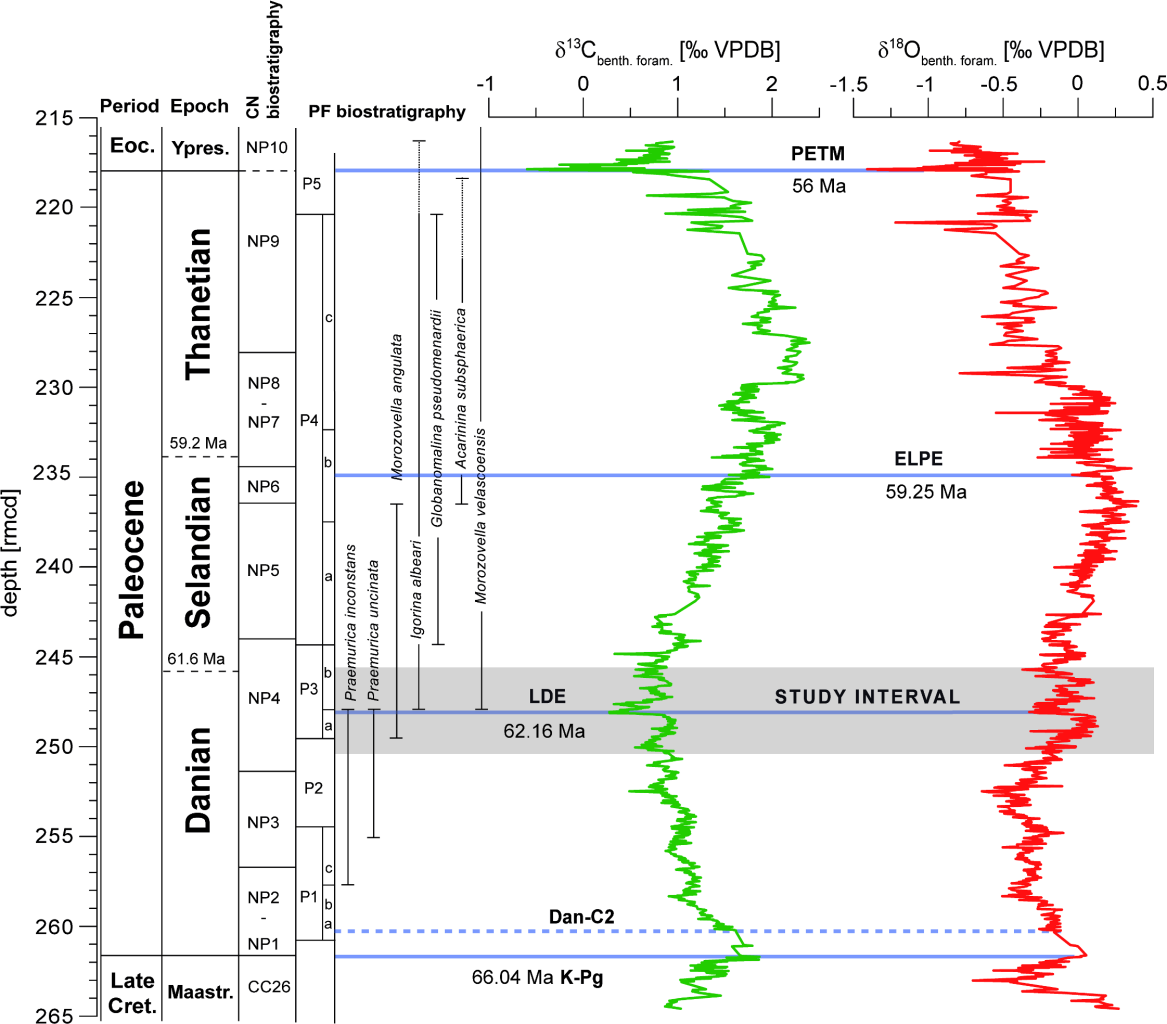
Stratigraphy, δ^13^C and δ^18^O measurements of benthic and planktic foraminifera on ODP Sites 1209: δ^13^C (green) and δ^18^O (red) in a long-term benthic *Nuttallides truempyi* record (plotted against rmcd [18]). Period, epoch, calcareous nannofossil, age and planktic foraminiferal biostratigraphy are from [18, 26], isotope data are adopted from [18]. Absolute ages given are based on [GTS 2012].

The LDE is characterized by a prominent negative δ^13^C excursion of at least 0.7‰ in different marine settings like the southern Tethyan shelf (Egypt [17]), the northern Tethys (Bjala, Bulgaria [19]), the eastern North Atlantic (Zumaia, Spain [23]), and the Pacific Ocean [18]. Moreover, the LDE is identified by the most negative δ^13^C values for the entire Paleocene, thus, representing an extreme position in the secular changes of the global carbon cycle [18] (Fig 1). Similar to other Paleogene hyperthermals the negative δ^13^C excursion might be attributed to the addition of huge amounts of ^13^C depleted carbon to ocean and atmosphere. Accompanied warming might be explained either by the possibility of high atmosphere greenhouse gas concentrations similar to the PETM or increased insolation due to the orbital constellation (Pc_405_10) [20] during the upper Chron C27n. Further, the LDE falls within a time interval with an increase of oceanic spreading rates and volcanic activity along the SE Greenland margin [24, 25].

**Fig 2:**
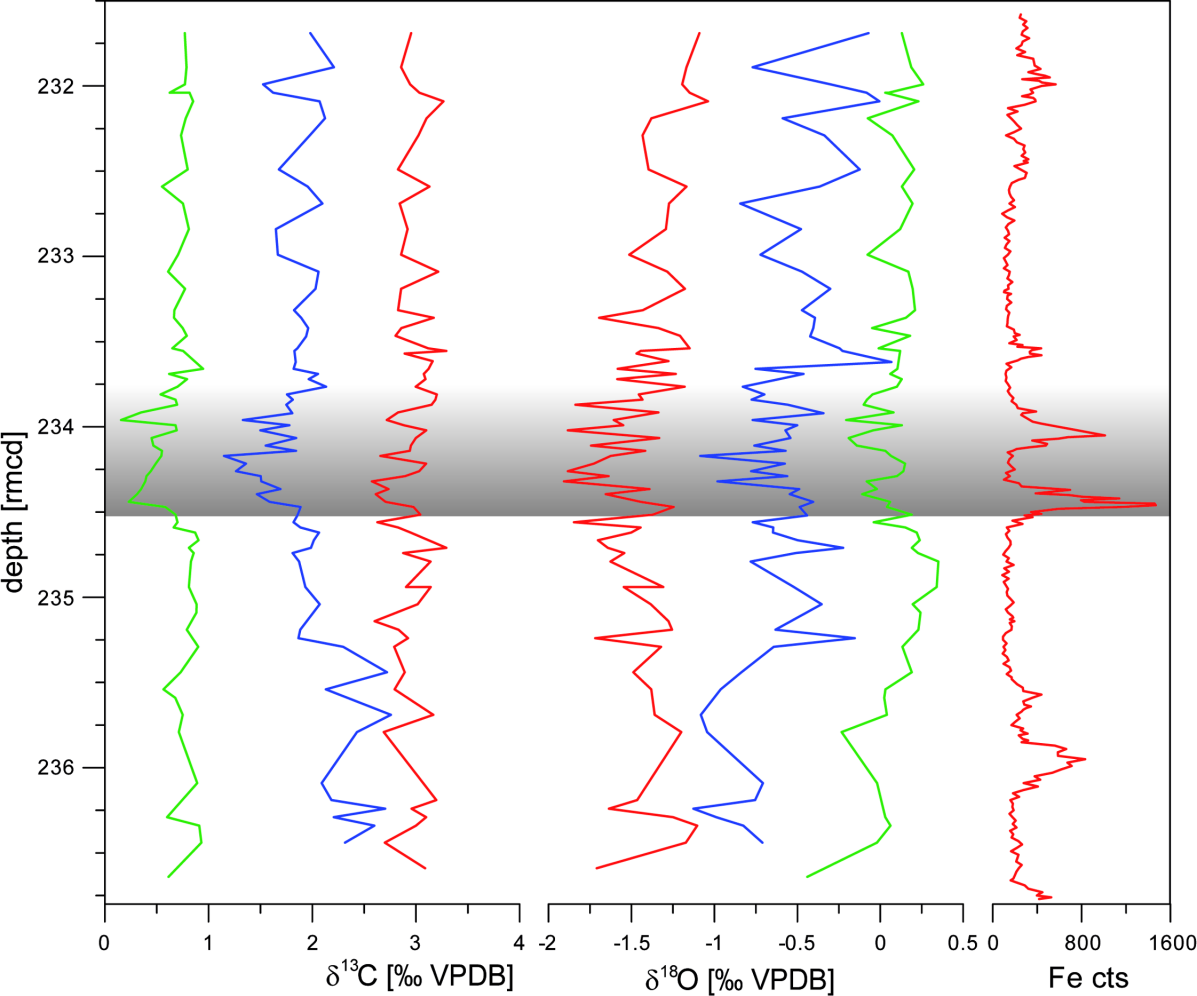
A) and B) δ^13^C and δ^18^O of benthic (green), planktic subsurface (blue) and surface (red) foraminifera in comparison to C) XRF measured Fe counts for chemostratigraphic correlation. The LDE is marked in grey, stable isotope data can be found in S2 Table.

In various Ocean Drilling Program (ODP) cores this event is marked by two prominent peaks in Fe intensities based on XRF core scanning (Fig 2) and magnetic susceptibility. According to orbital tuning the two LDE peaks correspond to two short eccentricity cycles suggesting a total duration of 190–200 kyr [19, 24] (Fig 2).

Due to the supra-regional nature of the LDE, the associated paleoenvironmental changes (e.g. oligotaxic benthic foraminiferal assemblages, poor oxygenation of the seafloor on the Southern Tethyan shelf) that resemble those of the PETM [17, 27, 28] and benthic foraminiferal δ^18^O data from nearby ODP Site 1209 [18] suggesting a bottom-water temperature rise of ~2°C (Fig 1), it has been hypothesized that the LDE might represent a Paleocene hyperthermal. In addition, biotic changes are associated with the LDE interval. Similar to the PETM at the C27n-C26r transition major turnovers of land mammals have been observed in China and North America [29, 30]. In the marine realm the LDE coincides with an interval of major diversification events in calcareous plankton groups suggesting re-organization of the surface oceans ecosystem. This also includes the evolutionary changes of the muricate *Acarinina* and *Morozovella* lineages in planktic foraminifera (e.g. [9, 31]), and a major radiation event of fasciculithids (e.g. [32–34]). However, all these studies are based on rather long-term records with a limited temporal resolution. So far, no detailed planktic foraminiferal data are available for the LDE from the deep-sea. Here we present the first high-resolution planktic foraminiferal faunal data across this event from ODP Site 1210 (Shatsky Rise).

Since planktic foraminifera are an important component of the marine food web as primary or secondary consumers they are highly sensitive to paleoceanographic changes, and should respond to environmental perturbations (e.g. [35]). In this paper we investigate for the first time changes in the community structure of planktic foraminiferal assemblages as a response to environmental changes during the LDE. Further, we test the hypothesis whether the LDE represents a transient warming event by analyzing δ^18^O and δ^13^C of benthic and planktic foraminifera, in order to gain insight into the evolution of the vertical ocean structure and other paleoenvironmental parameters. We further assess the degree of CaCO_3_ dissolution related to the LDE by analyzing the sedimentary CaCO_3_ content, percentage planktic foraminifera (%P), coarse fraction, and employ a planktic foraminiferal fragmentation index.

### 1.1 Geological setting

**Fig 3:**
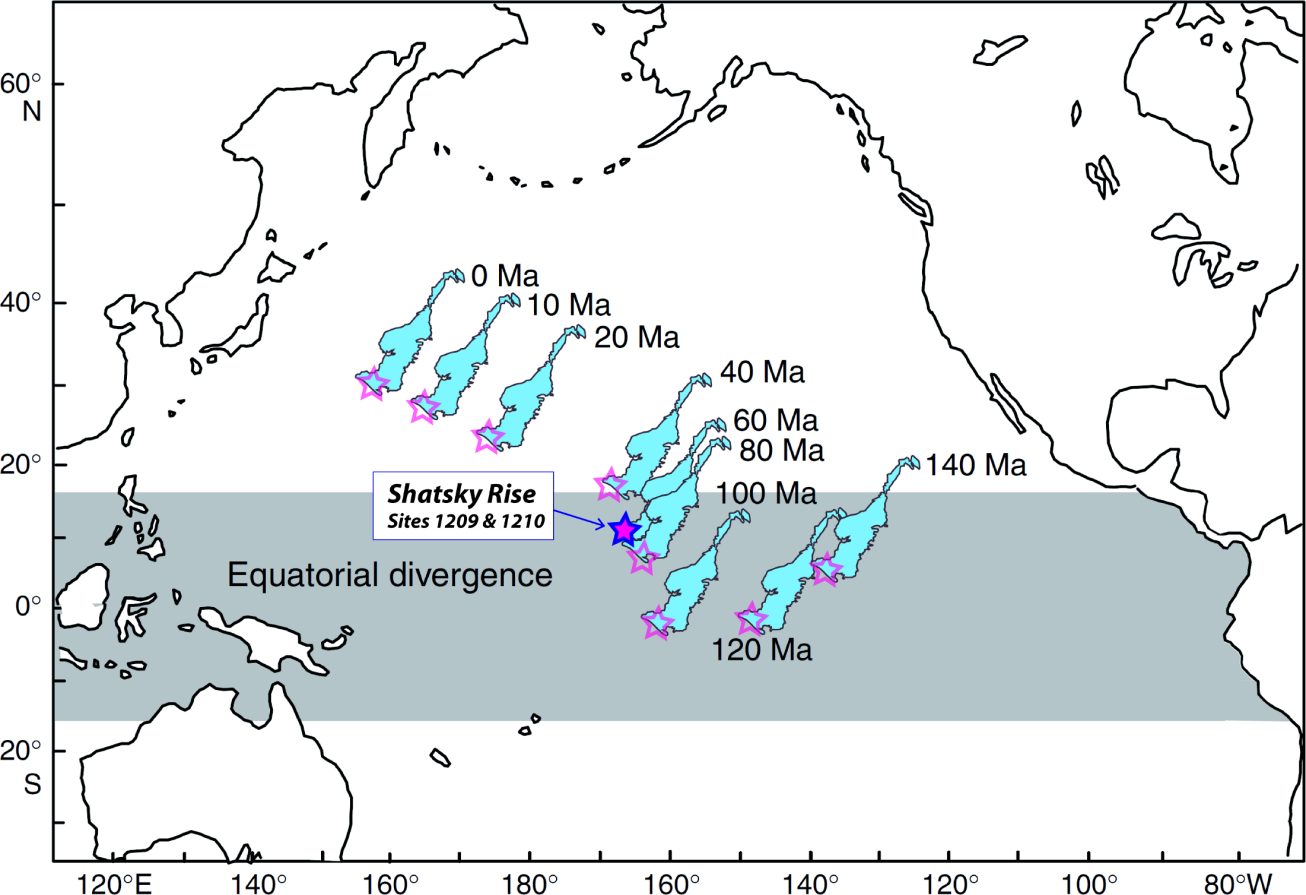
Pacific centered map of the Shatsky Rise (amended after [39]). ODP Sites 1209 and 1210 are marked by purple stars. Due to plate tectonic movements, the Shatsky Rise Plateau shifted north-westwards over the last 140 Myr. The blue framed star shows the studied site during the middle Paleocene (~60 Ma).

Shatsky Rise Plateau is a large igneous province situated on a triple-junction in the Pacific Ocean, formed in Late Jurassic to Early Cretaceous times (149–135 Ma) [36] (Fig 3). The plateau was then located close to the equator and drifted to the Northwest with changing velocities during the Cretaceous and the Paleogene. Shatsky Rise possibly crossed the equator during the Maastrichtian. Rates of subsidence and drifting were faster during the Jurassic-Cretaceous boundary interval and slowed down with time [11].

ODP Leg 198 Site 1210 (lat: 32.2235°N, long: 158.2594°W) is situated on the southern part of the Shatsky Rise Plateau and was drilled in 2001 at a water depth of 2573 mbsl (meters below sea level). Paleodepth has been estimated as upper abyssal to lower bathyal [11]. Neighboring ODP Site 1209 likely rose from a paleodepth of ~2500 mbsl to 2387 mbsl today [37], which suggests ~2700 m as paleodepth for Site 1210 assuming a uniform uplift of the plateau [38].

### 1.2 Lithology

**Fig 4:**
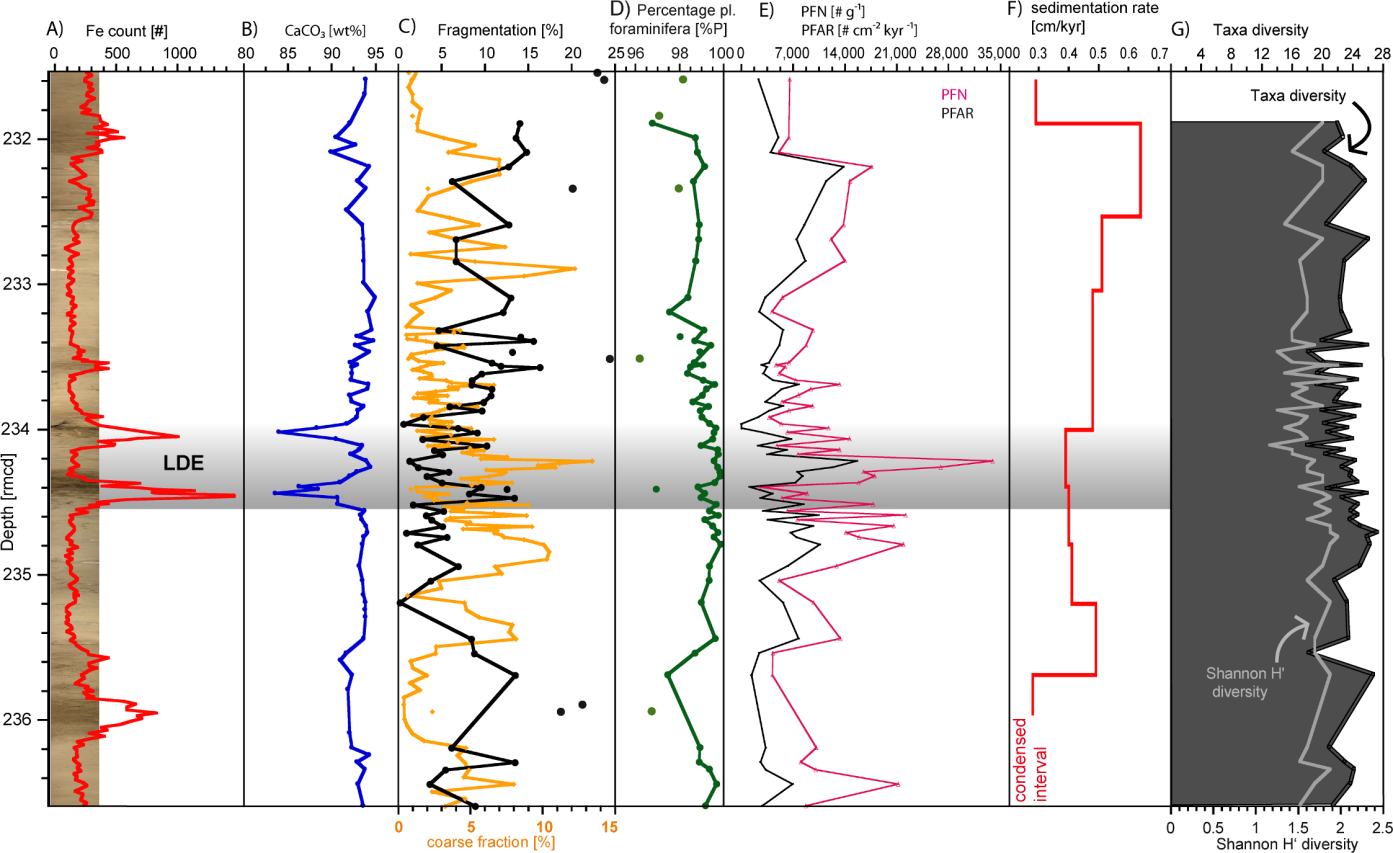
Sedimentary and other parameters of Site 1210. The LDE is marked in grey, data available in S4 Table. **A)** Biostratigraphy and Fe XRF core scanning data overlying the sediment core photo. The two main peaks mark the LDE and are used for stratigraphic correlation. The LDE interval covering the XRF Fe peaks are marked by a blue bar. The misfit between XRF core scanning Fe peaks and lithology is most likely an artifact of core expansion in storage. **B)** CaCO_3_ data. **C)** Coarse fraction (orange) and fragmentation (black) show opposing trends. **D)** Percentage of planktic foraminifera [%P] (green) shows only little variation below 100% with minima close to Fe maxima. For **C)** and **D)**: Data points marked as single symbols represent samples that have been excluded from faunal assemblage analyses due to potential diagenetic alteration. **E)** The amount of planktic foraminifera per g sediment (PFN [# g^-1^], pink) has a minimum close to the first LDE peak and a maximum shortly thereafter, however, the variability of planktic foraminiferal accumulation rates (PFAR, black, [# cm^-2^ kyr^-1^]) is much lower than the absolute abundance of planktic foraminifera per gram sediment. **F)** Sedimentation rate according to [24] based on the presented cyclostratigraphy therein. **G)** Simple diversity (grey) and Shannon H’ diversity (black line with a dark grayish background) both indicate a slight decrease during the LDE interval.

The more than 100-m-thick sedimentary succession of Paleogene age (base Paleocene to early Oligocene) at Site 1210 shows in general higher carbonate values and offers shades of orange and yellowish brown nannofossil ooze, nannofossil ooze with clay and minor amounts of clay with nannofossil ooze (Fig 4, S4 Table). A remarkable similarity in the lithologic record between Sites 1209 and 1210 seems to be evident [39]. The study interval at ODP Hole 1210A covers 5.1 meters of core 23H sections 1–4 (231.4–236.6 rmcd, revised meters composite depth, [40]). Two prominent dark brown horizons are intercalated into the monotonous calcareous ooze sequence representing the Latest Danian Event (233.4–234.6 rmcd). This key interval is about 0.5 m thick. Below the first event bed at 243.4 rmcd the color is darker and it becomes much lighter above the LDE. In addition to these two prominent layers, three less pronounced darker beds are intercalated into this succession (at ~236 rmcd, at ~ 233.6 rmcd right above the LDE, and at 232 rmcd below it; Fig 4, S4 Table).

### 1.3 Stratigraphy

Synonymously to the Latest Danian Event (LDE) [17] the term “Top Chron C27n Event” is often used [18, 19, 24] indicating the magnetostratigraphic position of this event as observed at stratigraphically well-calibrated ODP Site 1262 (Walvis Ridge, [24]) and Zumaia (Spain) [19, 23]. The latter represents the Global Stratotype Section and Point (GSSP) for both base and top of the Selandian Stage [41]. However, no reliable magnetostratigraphy exists for the study interval at Site 1210, but supra-regional chemo- and cyclostratigraphic correlation supports the same stratigraphic position of the LDE at Sites 1209 and 1210 [24]. In many deep-sea sections the LDE is characterized by two distinct peaks in magnetic susceptibility and Fe XRF core scanning data, which are paralleled by a prominent (~0.7 ‰) negative CIE in benthic foraminifera [18, 26]. The two prominent Fe peaks of the LDE have also been observed at ODP Site 1210 (Figs 2 and 4). The very similar pattern in XRF measurements allows to apply the astronomically-tuned 1209 age model to Site 1210 [24].

Recent studies propose that the LDE coincides with 405 kyr-precession cycle 10 [20, 42]. Based on such a correlation we infer that the study interval covers ~900 kyr and pinpoint the largest and lowest LDE Fe peak at 234.45 rmcd to an absolute age of 62.18 Ma, just before the Danian-Selandian boundary at 61.61 Ma as suggested by [20] and [43]. Biostratigraphically, the LDE is positioned with its lowest XRF Fe peak close to the P3a–P3b planktic foraminiferal subzonal boundary and within the NTp7B and NP4 nannofossil zones [44].

## Methods

Seventy-three samples were taken at a resolution of 2 to 15 cm with the highest resolution across the assumed LDE. All samples were approved and provided by the International Ocean Discovery Program (IODP) Gulf Coast Repository (College Station, Texas, USA). No further specific permissions were required for the studied location. The performed study did not involve endangered or protected species. Sample material is stored at the geological collections of the Institute for Geophysics and Geology, University of Leipzig.

The samples were studied with respect to δ^13^C and δ^18^O of planktic and benthic foraminifera, CaCO_3_ content, planktic foraminiferal assemblages, percentage of planktic foraminifera and other parameters like fragmentation, coarse fraction, absolute abundances per gram sediment (planktic foraminiferal number, PFN). In addition, planktic foraminiferal accumulation rates (PFAR) have been calculated using the published age model [24] and shipboard dry bulk density data [11].

Sediment samples were oven-dried at 40°C for at least 48 hours. The sediment was weighed and soaked in tap water. In some samples, the sediment disintegrated within a few minutes and was processed immediately. Samples were washed through a 63-μm-mesh sieve, transferred onto filtering paper with deionized water and a splash of acetone, and then gently dried for at least 48 hours at 40° C.

Coarse fraction is calculated by:

$$CF=\left(\frac{dry\;weight\;residuum>63\mu m}{dry\;weight\;total\;sample}\right)\times 100\;[\%]$$ (1)

Percentages of planktic foraminifera (%P) were counted from the >63 μm sieve size fraction and are primarily used to assess dissolution intervals since planktic foraminifera are more prone to dissolution than benthic foraminifera (e.g. [45]). For faunal analysis, the >125 μm fraction was split into proportions containing 300–600 planktic specimens with a microsplitter and identified under a Zeiss Stemi 2000-C binocular microscope at 50☓ magnification. Planktic foraminiferal taxonomy follows [46] and [47].

The fragmentation index was calculated as follows, counting tests larger than half as complete tests and tests smaller than half as fragments in the >125 μm size fraction [48]:

$$F=\frac{quantity\;fragments}{quantity\;fragments+quantity\;complete\;tests}\times 100[\%]$$ (2)

Absolute abundances of planktic foraminifera (PFN, [# g^-1^]) were calculated using dry weight of the sample, number of counted specimens (>125μm) and the applied split-factor. Planktic foraminifera accumulation rate (PFAR) was calculated by dry density (DBD, [11]) multiplied with linear sedimentation rate (LSR) as well as abundance per g (PFN):

$$PFAR=DBD\times \mathrm{LSR}\times \mathrm{PFN}\;[\#\;{\mathrm{cm}}^{-2}\;{\mathrm{kyr}}^{-1}]$$ (3)

The diversity of planktic foraminiferal assemblages was characterized by species richness (S) and Shannon heterogeneity (H’). The latter one was calculated by using an information function [49] as follows:

$$H’=-\sum_{i=1}^{k}p_{i}\times \ln\;p_{i}$$ (4)

with p_i_ as the proportion of individuals belonging to the *i* ^th^ species in the dataset of interest.

For δ^13^C and δ^18^O measurements 3 to 7 specimens were reacted with 100% phosphoric acid at 75°C using a Kiel IV online carbonate preparation line connected to a MAT 253 mass spectrometer. Reproducibility was checked by replicate analysis of laboratory standards and was better than 0.05‰ and 0.06‰ for δ^13^C and δ^18^O, respectively. Planktic specimens were picked from the 250–355 μm size fraction. Where this did not yield sufficient material for the analysis, 180–250 μm was used additionally. Benthic foraminifera were mostly picked from 125–180 μm.

We generated stable isotope (δ^13^C, δ^18^O) records of the best preserved planktic and benthic foraminifera. Bottom water conditions were estimated from analyses of epibenthic *Nuttallides truempyi* and in some samples barren of *N*. *truempyi*, *N*. *umbonifera* or *N*. *truempyi/umbonifera* was used. A t-test was performed on δ^13^C and δ^18^O of benthic, subsurface- and surface-dwelling foraminifera comparing the isotope values before, during and after the LDE. Subsurface conditions are inferred from analyses of *Parasubbotina pseudobulloides/variospira* and *P*. *varianta*, while surface water conditions were determined using *Morozovella angulata*. Further multiple-test measurements were carried out on *Igorina albeari*, *M*. *velascoensis* and *M*. *conicotruncata*, *Globanomalina chapmani*, *G*. *imitata* and *Acarinina strabocella*, *Praemurica praecursoria*, *Pr*. *uncinata* and *Pr*. *inconstans* in order to better constrain their depth habitat. Relative temperature changes were estimated based on the calibration equation of [50]. Calcium carbonate values are derived from measurements carried out with an Elementar III (VARIO Corp.) CNS analyzer.

For the statistical analysis, we used the relative abundances of all samples that have not been excluded due to a potential preservational bias (n = 59), see explanation below. In order to analyze changes in the assemblage composition non-metric multidimensional scaling (NMDS) was applied, which is one of the most widely used ordination techniques and one of the most robust unconstrained ordination methods in community ecology (cf. [51, 52]). We used the “vegan package” for R to run ecological multivariate statistics. Environmental vectors as represented by the δ^13^C and δ^18^O of benthic, subsurface- and surface-dwelling foraminifera, and sedimentary CaCO_3_ have been fitted onto the NMDS ordination. For this the ‘envfit’ function of the vegan package was used in order to test if the vector projections correlate with the gained faunal patterns.

## Results

### 3.1 Carbonate content and dissolution sensitive parameters

CaCO_3_ values range from ~84 to 95 wt%, with minima within the two LDE beds with values around 84 wt%. The background values (~94 wt%) are rather uniform (Fig 4, S4 Table). Other carbonate dissolution sensitive parameters (Fig 4) are coarse fraction (CF), fragmentation of planktic species (%F), percentages of planktic species (%P), absolute abundance of planktic foraminifera (>125 μm) per gram sediment (expressed as planktic foraminifera number, PFN), connected to planktic foraminifera accumulation rate (PFAR, [# cm ^-2^ kyr ^-1^]), and diversity.

CF percentages vary between 1 and 14% below the LDE, displays three peaks or outliers of 7 to 11% and a minimum at 234.4 rmcd. The fragmentation index (%F) ranges between 0 and 24%, and shows a reversed trend to CF with a maximum of 18% coinciding with the position of the minimum in CF (Fig 4).

PFN (Fig 4) increases to ~34,000 # g^-1^ between the lowest and second LDE peak, which is also the highest value within the study interval. A strong minimum is visible concurrent with the lowest LDE iron peak. The background signal varies between 4,000 and 20,000 specimens. PFAR (Fig 4) ranges from 1,115 to 15,612 # cm^-2^ kyr^-1^ and is much smoother than PFN pointing to less substantial variations in the numbers of planktic foraminiferal tests. No significant correspondence to the LDE has been observed there.

Percentages of planktic foraminifera (%P, >63 μm, Fig 4) vary between 96.5 and 100% with three distinct minima: (1) below the LDE (~235.7 rmcd), (2) within the lowest LDE peak (~234.4 rmcd), and (3) at the top of the study interval (~231.9 rmcd). Apart from the drop in one sample within the lowest LDE peak, %P is rather constant with ~ 98.5–99.5% across the entire LDE interval.

Simple diversity (S) varies between 13 and 23 species per sample (Fig 4), Shannon diversity (H’) ranges from 1.64–2.47 with an average of 2.08. The lowest diversity for both scales is found at the beginning of the second LDE Fe peak and stays low until 233.5 rmcd after the LDE. Diversity mean is slightly higher before than after the event by 0.1 (2.16 before, 2.09 during and 2.06 after the LDE).

### 3.2 Foraminiferal stable isotopes (δ^13^C, δ^18^O)

#### 3.2.1 High resolution records of planktic and benthic foraminifera

All three groups, surface dwelling (*Morozovella angulata*, *M*. *praeangulata*), subsurface dwelling (*Parasubbotina pseudobulloides*, *P*. *varianta*) and benthic foraminifera (*Nuttallides truempyi*, *N*. *umbonifera*, *N*. *umbonifera-truempyi* intermediate) show negative excursions for both δ^13^C and δ^18^O of different amplitudes at the lowest LDE peak (Fig 2, S2 and S3 Tables).

**Fig 5:**
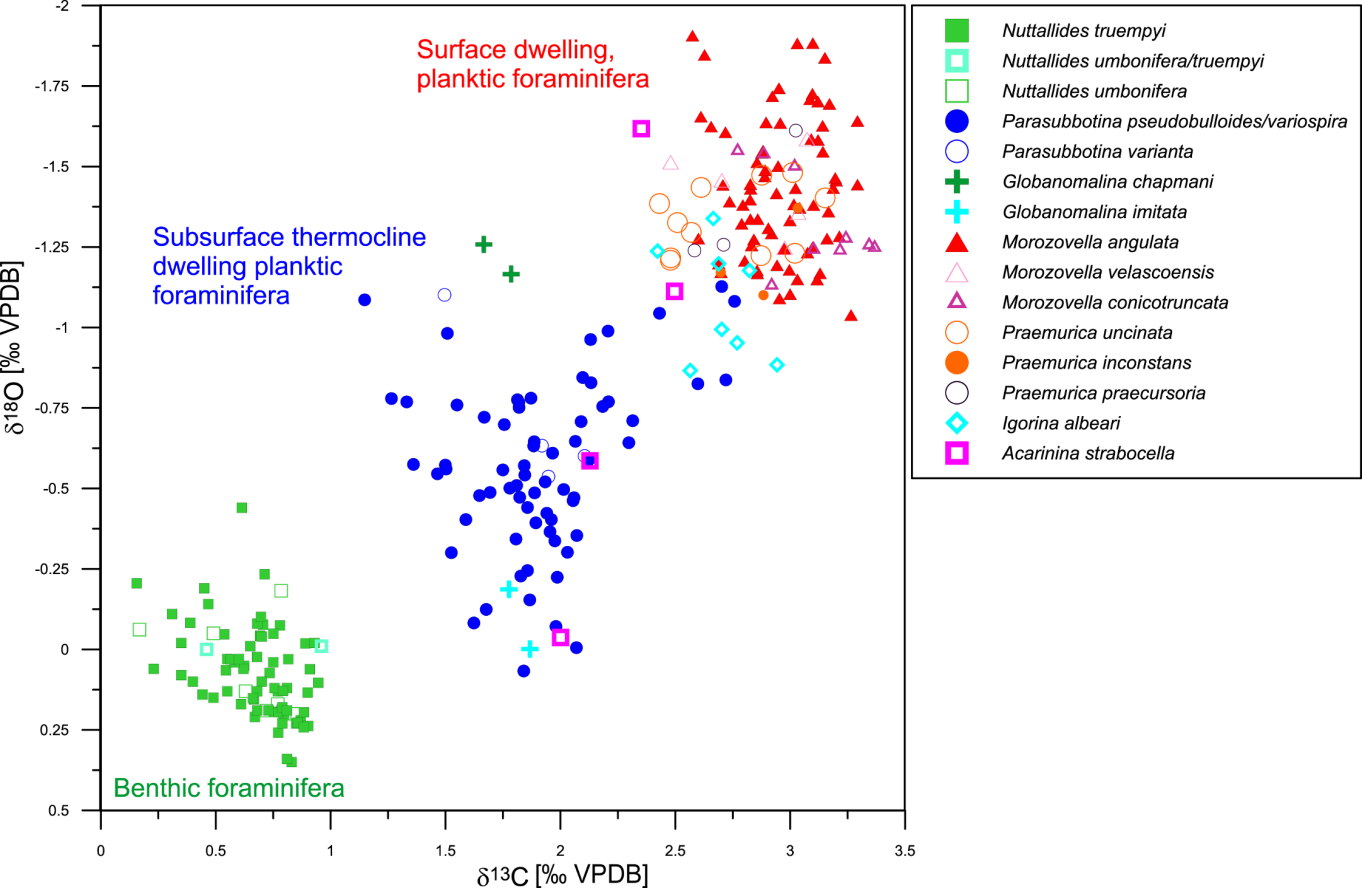
δ^13^C–δ^18^O plot of foraminiferal isotope measurements. Distinct clusters separate epibenthic (*Nuttallides truempyi*, *N*. *umbonifera* and intermediate form), planktic subsurface (e.g. *Parasubbotina pseudobulloides/variospira*, *P*. *varianta*) and surface dwelling taxa (e.g. *Morozovella angulata*). Besides these, isotopic signatures of other taxa were measured to better understand their depth-habitat and paleoecology. Data available in S2 and S3 Tables.

Species-specific measurements on surface dwelling taxa (*Morozovella angulata*, *M*. *praeangulata*) range from 2.55 to 3.3‰ for δ^13^C and from -1.9 to -1.2‰ for δ^18^O (Figs 2 and 5). Subsurface dwellers (*Parasubbotina pseudobulloides*, *P*. *varianta*) display values between 1 and 3‰ for δ^13^C and between -1.1 and -0.4‰ for δ^18^O, while δ^13^C and δ^18^O values of benthic foraminifera are of 0.2 to 0.9‰ and -0.1 to 0.35‰, respectively.

At the base of the lowest LDE peak, δ^13^C drops in planktic surface dwellers by ~0.7‰ (3.25 to 2.55‰), whereas subsurface ones decrease by ~0.9‰ (2.1 to 1.2‰) and benthic by ~0.6‰ (0.8 to 0.2‰; Fig 2). Accordingly, a negative carbon isotope excursion (CIE) coincides with the lowest LDE Fe peak. For surface dwelling taxa, δ^18^O decreases by ~0.6‰ (-1.3 to -1.9‰), at the subsurface ocean by ~0.5‰ (-0.5 to -1‰) and on the sea-floor (benthic foraminifera) by ~0.4‰ (0.3 to -0.1‰; Fig 2). δ^18^O and δ^13^C show partly significant differences between before, during and after the event as tested by the two-sided t-test (cf. Table 1 t-test). The development for the δ^13^C record is generally more significant than for δ^18^O except from *M*. *angulata*, which varies within a smaller range. δ^18^O shows no or low significant difference for *P*. *pseudobulloides* and *N*. *truempyi* between below the event and during the LDE. The t-test results of *P*. *pseudobulloides* might be influenced by the wide scatter of isotope values influencing the mean, which is compared in the analysis. Same sample segments for the intervals before, during and after the LDE were used for the NMDS analysis.

**Table 1:** T-test p-values numbers marked in bold indicate numbers that are significant on the 95% confidence limit.

	*M*. *angulata*		*P*. *pseudobulloides/variospira*		*N*. *truempyi*	
	Post-event vs. LDE	LDE vs. Pre-event	Post-event vs. LDE	LDE vs. Pre-event	Post-event vs. LDE	LDE vs. Pre-event
N	23/20	20/25	23/20	20/25	26/21	21/24
p-values T-testδ^13^C	0.07128	0.54873	**0.00020459**	**4.3639E-07**	**1.3505E-07**	**9.523E-08**
p-values T-test δ^18^O	**9.7945E-06**	**0.03424**	**0.0003105**	0.37363	**0.0046499**	0.099652

#### 3.2.2 Isotope results of selected planktic foraminifera species

δ^13^C and δ^18^O values of *M*. *occlusa* are similar to coexisting *M*. *velascoensis* and *Acarinina mckannai*. *Morozovella velascoensis* and *M*. *conicotruncata* generally appear in the same range as *M*. *angulata*, but both with higher δ^18^O values. *Morozovella velascoensis* values are close to *M*. *angulata* while *M*. *conicotruncata* shows a different course for δ^13^C towards heavier values after the LDE CIE.

*Acarinina strabocella*, measured on only four samples, show lower δ^18^O before than after the LDE and higher δ^13^C before than after it. Measurements on *Praemurica inconstans* and *P*. *praecursoria* show low δ^18^O values compared to results of *P*. *uncinata*.

*Igorina albeari* reveals values between those of surface and subsurface dwellers but slightly closer to the surface ones (Fig 5). The temporal course continuously parallels *M*. *angulata* with lower δ^13^C and higher δ^18^O.

### 3.3 Planktic foraminiferal assemblages

**Fig 6:**
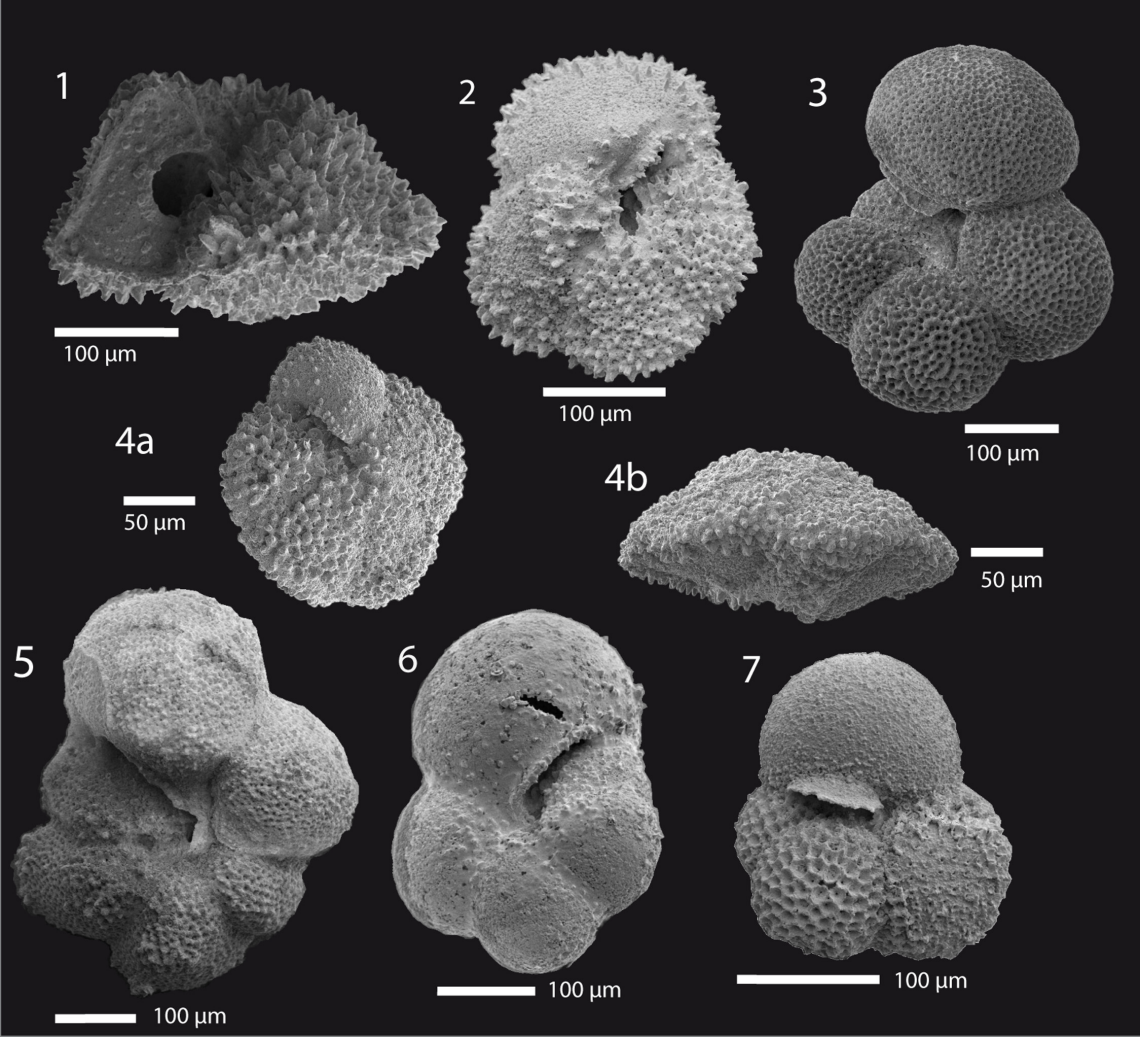
Scanning electron microscope images of planktic foraminifera. **1:** *Morozovella angulata*, lateral view (Sample 1210A-23-3, 0–1.5 cm); **2:** *Morozovella aequa*, umbilical view (1210A-23-3, 37.5–39 cm); **3:** *Parasubbotina variospira*, umbilical (1210A-23-3, 30–31.5 cm); **4a+4b**: *Igorina albeari* umbilical/lateral (1210A-23-1, 90–92 cm); **5:** *Praemurica uncinata*, umbilical (1210A-23-3, 52.5–54 cm); **6:** *Globanomalina chapmani*, umbilical (1210A-23-1, 85–87 cm); **7:** *Subbotina triangularis*, umbilical (1210A-23-3, 52.5–54 cm).

**Fig 7:**
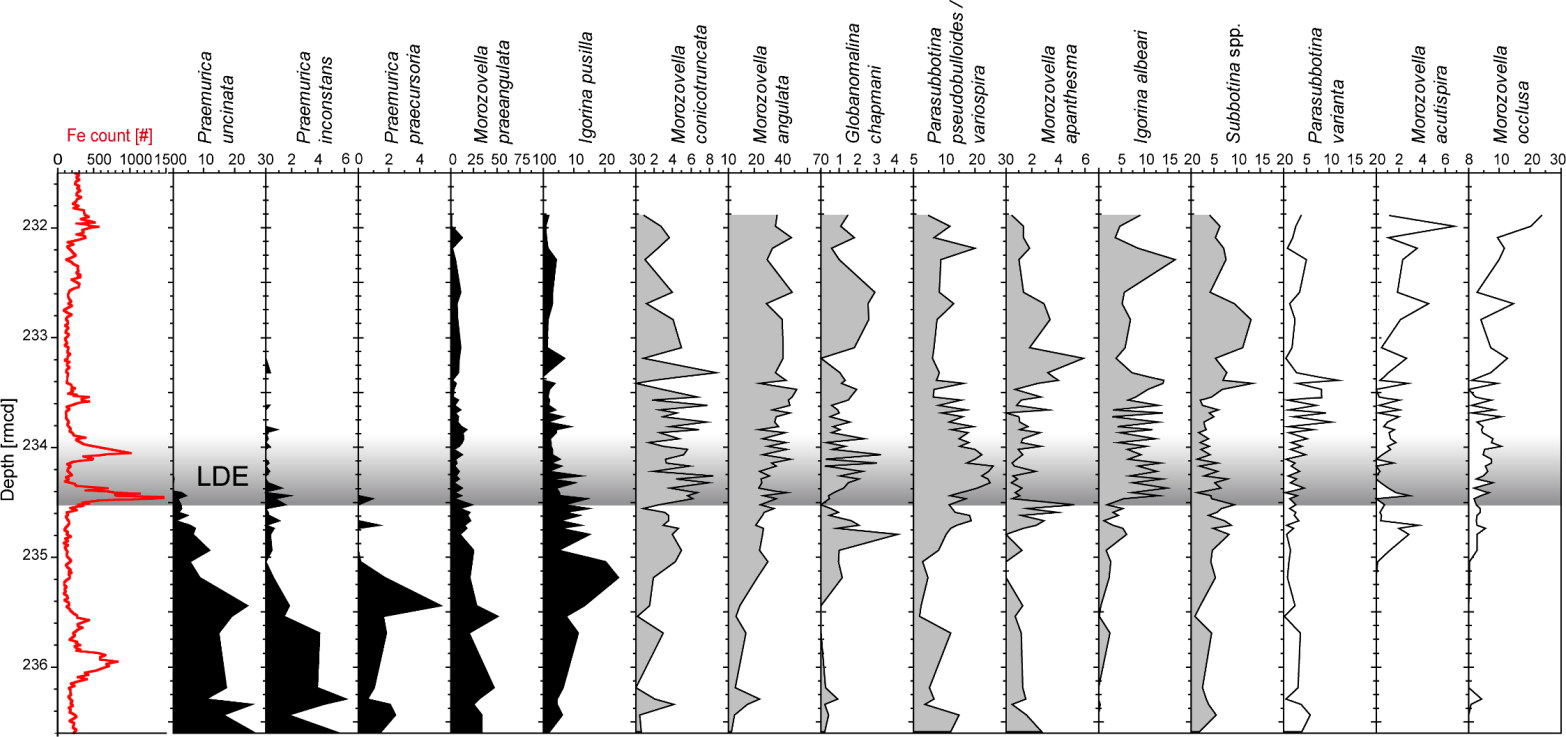
Species abundance. Fe XRF core scanning data for chemostratigraphic correlation and species abundance data (percentages). Species are sorted according to the stratigraphic order of their abundance maxima. The LDE is marked in grey, data available in S5 Table.

Planktic foraminiferal faunas (Figs 6 and 7) are dominated by nine taxa that make up ~86% of the total assemblage. Here they are described in the stratigraphic order of their abundance maxima:

*Praemurica uncinata* is quite common (c. 26% at 236.6 and 235.4 rmcd) before the LDE, but virtually disappears at the base of the LDE. A similar trend is observed for the less abundant *P*. *inconstans* and *P*. *praecursoria*.

*Morozovella praeangulata* has a strongly muricate peripheral margin which is rounder than that of *M*. *angulata*, but *M*. *praeangulata* has in general a similar appearance [47, 53]. *Morozovella praeangulata* decreases steadily from 35 to 10% abundance below the LDE and shows a massive break-down around the first LDE horizon at 234.5 rmcd.

*Igorina pusilla* has its main peak with 24.5% before the LDE (235.2 rmcd) to decrease at the LDE onset. The abundance of *I*. *pusilla* oscillates strongly, as does *I*. *albeari*. It decreases from 15% (234.8 rmcd) to about 2% and almost disappears shortly thereafter, displaying a similar course as *M*. *praeangulata*.

*Morozovella conicotruncata* shows a constant rise (up to 5%) between 236.5 to 234.8 rmcd. Its abundance drops to 0.8% just below the LDE and oscillates between 1 and 8% at 234.4 to 233.5 rmcd. Abundances above the LDE vary between 1 and 5% with one exception at 8.7%.

*Morozovella angulata* increases steadily from 2 to 40% over the study interval with peaks of 52% above the second LDE horizon at 233.5 rmcd.

A few specimens of *Globanomalina chapmani* appear below 235.5 rmcd. The abundance rises to 5% right below the LDE and strongly decreases afterward with varying abundances of less than 3%.

In this study *Parasubbotina variospira* and *P*. *pseudobulloides* have been lumped together for assemblage counting due to an inconsistent taxonomic definition and a similar morphology. They have a slight variation in trochospiral chamber arrangements with a wider umbilicus of *P*. *variospira* without a distinct threshold between both species [47]. Abundance patterns of *Parasubbotina pseudobulloides/variospira* present a belly-shaped tipping 26% (234.2 rmcd) with 5 to 10% below and above the LDE.

*Morozovella apanthesma* only appears in small numbers but throughout the entire study area. Peaks are at 234.5 rmcd which rises just before and with the lowest LDE peak and at 233.4 rmcd above the event with a maximum of 6%.

*Igorina albeari* is the marker species for the subzone P3b [54, 55] and occurs throughout the entire study interval, which implies that the base of P3b is well below the LDE at Shatsky Rise. It starts with an abundance of 0.2–6%, to rise with the onset of the LDE at 243.5 rmcd to 14.8%.

*Subbotina* spp. shows a slight increase before the LDE with a maximum of 9% to decrease during the event down to 3–5% and recover afterwards to ~12%.

Relative abundance of *P*. *varianta* varies between 2 and 4% with a maximum during the LDE. Highest values of ~12% are observed directly above the LDE.

*Morozovella occlusa* is distinguished from *M*. *acutispira* by its lower number of chambers in the final whorl (generally four to six, maximum eight in *M*. *occlusa;* eleven to twelve in *M*. *acutispira*). *Morozovella occlusa* is suggested to be closely related to *M*. *angulata* [47, 56]. *Morozovella acutispira* appears for the first time at 235 rmcd below the LDE to slowly rise in abundance to up to 4% and above it abundance rises to 7%. A similar trend is shown by *M*. *occlusa* as values rise above 5% 15 cm below the LDE peaks at 235 rmcd for the first time. During the LDE, it has its highest abundance in the upper part with 11% (234 rmcd) and low numbers at the top of the LDE (233.5 rmcd) after which this taxon visibly increases in abundance from 5% (few samples) to 25% (233.4 rmcd and upwards). Results from multivariate statistics (NMDS) of the faunal data are discussed in section 4.5.

## Discussion

### 4.1 Biostratigraphic implications

According to planktic foraminifera biostratigraphy the LDE has to date usually been placed at or just above the P3a–P3b subzone boundary [17, 18, 44] as defined by the first appearance datum of *Igorina albeari* [54, 57], however, there is still a discrepancy in the definition on this taxon (cf. [44, 58, 59]). *I*. *albeari* might have closely related species, as determined by [59], but here we strictly follow the taxonomic description given by [47]. Results from our study suggest that *I*. *albeari* (sensu [44]) already appears consistently at least 400 kyr below the LDE (Fig 7). Thus, we suggest that at Shatsky Rise the first common abundance of *I*. *albeari* marks the onset of the LDE at 234.4 rmcd rather than a simple first (rare) occurrence. Therefore, the P3a–P3b boundary is suggested to be well below the LDE. Based on this early occurrence at Shatsky Rise we tentatively propose that this species may have evolved from *I*. *pusilla* in the Pacific and appears later in other ocean basins. *G*. *pseudomenardii* (defining the base of Zone P4) was observed in rare abundances at a lower stratigraphic position than expected and may have, thus, possibly been overseen in other biostratigraphic studies or alternatively appears earlier in the Pacific Ocean. Further, specimens with slight morphological differences to *G*. *pseudomenardii* were found and named *G*. cf. *pseudomenardii* as they do not allow for an unequivocal identification of this species. However, these findings need to be confirmed at other deep-sea sites.

### 4.2 Carbonate preservation

Changes in foraminiferal test preservation due to species-specific dissolution susceptibility can alter the assemblage composition and, thus, bias the ecological and environmental interpretation of faunal data (e.g., [60–63]). Moreover, dissolution, but specifically recrystallization can cause changes in the geochemical composition of foraminiferal calcite.

In this study we assessed a potential preservational bias of the faunal data by using, in addition to visual criteria, a combined approach comprising coarse fraction data (CF, >63 μm), absolute abundances of planktic foraminifera (PFN, >125 μm), %P (>63 μm), planktic foraminiferal fragmentation (%F, >125μm) and diversity changes (Fig 4). Eight samples were excluded from further interpretations based on this approach using a threshold of 18% for the fragmentation index since these high values are also accompanied by low values of both %P and CF and a lower planktic foraminiferal diversity pointing to a diagenetically controlled bias of the faunal composition (Fig 4). For comparison, planktic foraminiferal fragmentation during the PETM interval in Shatsky Rise Hole 1209B [49] displays background values of 10–20% and rise to a maximum of 45% during the PETM.

Visual observations of planktic foraminiferal assemblages reveal a general good preservation in nearly all samples. Foraminiferal tests are unfilled, primary features like pores, keel structures and openings are usually well developed as is evident from SEM images (Fig 6). Apparent dissolution features on tests such as missing chambers or broken walls have rarely been observed, but many specimens show indications of recrystallization, which is not surprising with background CaCO_3_ values of >90 wt%. The intervals with lower CaCO_3_ like the LDE beds contain relatively high abundances of dissolution-sensitive *Igorina albeari* [62, 63]. This shows that these samples are not significantly altered by dissolution. The brownish event beds of the LDE display slightly lower values of ~84 wt% CaCO_3_ (Fig 4).

Coarse fraction (CF) shows similar patterns to Site 1209, where the CF generally follows short-eccentricity cycles [18]. The highest CF peak occurs between the lowest and second LDE Fe peaks while minima correspond to each of these. Since the > 63 μm fraction consists almost exclusively of planktic foraminifera due to the absence of sand-sized terrigenous input in the central Pacific and negligible abundances of radiolarians, changes in the coarse fraction are predominantly controlled by planktic foraminiferal productivity, test fragmentation or CaCO_3_ dissolution. High intensities in Fe XRF core scanning in combination with low CF are suggested to be a further indication for enhanced dissolution at 232.00, 234.00, 234.40 and 235.95 rmcd (Fig 4).

While planktic assemblages, PFN and fragmentation were both from >125 μm size fraction, %P was analyzed on >63 μm. Using this size fraction probably leads to a higher amplitude of changes than expected for the >125 μm fraction for two reasons: (1) the smaller size fraction usually contains plenty of juveniles and more fragile taxa and therefore will be first fragmented, and (2) dissolution susceptibility is strongly species-dependent [50, 63]. Fragmentation of planktic foraminifera is widely used as an indicator for CaCO_3_ dissolution [64]. Planktic foraminiferal tests are usually more prone to dissolution or recrystallization than most benthic ones (e.g. [45, 60]).

General dissolution rankings of late Paleocene and early Eocene planktic foraminifera from Shatsky Rise and Allison Guyot [63] are inferred from laboratory experiments where the genus *Igorina* is considered to be more prone to dissolution than *Acarinina*, *Morozovella* and most *Subbotina*. In this study, the species *I*. *pusilla* and *I*. *tadjikistanensis* show a high dissolution-sensitivity. Opposed to that, faunal dominance of *Igorina* species was observed [21, 22] during the Early Late Paleocene Event (ELPE, 58.9 Ma, [21]) which is partly seen as a dissolution event [21]. It might imply that *Igorina* truly flourished during the ELPE and the species might still be prone to dissolution.

Sedimentation rates (SR; Fig 4) of Site 1210 (adapted from astronomical tuning [24]) show variations between 0.28 and 0.64 cm kyr^-1^ with an average of 0.44 cm kyr^-1^ during the short-eccentricity cycles (Pc_100_ 34–44). Due to the lack of terrestrial hinterland at Site 1210 the main sediment material originates from carbonate microfossils, whereas radiolarians as well as terrigenous dust and clays are only minor contributors to the sediment. Assuming a constant pelagic carbonate factory during the study interval the SR may reflect changes in the rates of carbonate dissolution (Fig 4) as is also suggested by the anti-correlation to short-eccentricity derived SR. The SR is lowest at the lowest and uppermost part of the studied interval with values between 0.2 and 0.35 cm kyr^-1^ [24]. Values are slightly decreasing during the LDE, which can point to a lower carbonate supply or increased dissolution, or both. This fact might be related to a lysocline shoaling during the LDE as observed during the PETM [8], and is supported by the dark event horizons, high XRF Fe counts and lower carbonate content. Planktic foraminiferal accumulation rate (PFAR) which also considers the sedimentation rates shows only minor changes that can be clearly attributed to the LDE (Fig 4).

Furthermore, the good correlation (R² = 0.59, N = 67, p<0.001, our data) between planktic proportion and fragmentation is considered as a useful indicator for carbonate dissolution [65]. The simultaneous trends of fragmentation increase and planktic proportion decrease (Fig 4) might therefore be also connected to lysocline variations. However, the overall correlation is somewhat lower than in [65] (R² = 0.77) from neighboring ODP sites over the entire Paleocene. Between 61.33–63 Ma, their R^2^ is even 0.965 (N = 5, p<0.001, calculated from published data [65]), which most likely results from a particularly small sample quantity. Based on this approach we assume that carbonate dissolution as a consequence of a lysocline rise during the LDE at Site 1210 might be minor compared to the ELPE and PETM.

To conclude, we suggest that the diagenetic alteration in terms of dissolution of the sample material is limited in the LDE. %P never drops below 95.5% and fragmentation never reaches values above 17% in the selected samples. This suggests only minor changes due to dissolution. Recrystallized tests are assumed to break much easier which might have also lead to enhanced fragmentation rates [66].

### 4.3 Foraminiferal stable isotope data

Test alteration due to secondary overprint is characterized by two successive stages after calcification: early diagenesis and diagenesis after burial (e.g. [67]). It is well known that diagenetic alteration of foraminiferal calcite has an influence on δ^13^C and δ^18^O values, specifically if recrystallization and precipitation of diagenetic calcite is involved [61, 68, 69]. While δ^13^C of recrystallized planktic foraminifera from deep-sea cores shows usually similar values to unaltered calcite [61], δ^18^O tends to increase due to the bottom-water signal recorded by precipitated secondary calcite during early diagenesis [61, 70]. Previous studies suggest that absolute δ^18^O values are biased by recrystallization, whereas trends and amplitude of changes are largely preserved [61, 71]. Calcite recrystallization would therefore rather damp the interpreted temperature change than amplifying it. It is further proposed that benthic foraminifers are probably less affected by diagenesis, therefore δ^18^O paleothermometry remains a valuable benchmark [61].

Even after picking the visually best preserved specimens for stable isotope analyses we cannot fully rule out recrystallization. Thus, we consider δ^13^C to be largely unaltered, whereas absolute δ^18^O values might have slightly changed, but we assume that the relative warming related to the LDE represents a primary signal as explained above. The reason for the generally noisier δ^18^O signal might be (1) the higher hydrographic variability of surface water masses compared to the deep-sea, (2) the fact that planktic foraminifera are more prone to diagenetic alteration specifically of absolute values of δ^18^O, or, most likely, a combination of both.

Benthic foraminiferal isotope data from Site 1210 are similar to those of Site 1209 [18]. However, the benthic foraminiferal δ^13^C excursion at the base of the LDE is better developed at Site 1210 (Fig 2). The CIE consists of a rather sharp base and an asymmetry that resembles the typical shape of the PETM CIE and contrasts the symmetric δ^13^C pattern known from early Eocene hyperthermal events like the ETM-2 or the MECO (e.g., [67, 72]). This shape may suggest a rapid injection of isotopically light carbon to the ocean as a trigger mechanism for the LDE and/or CaCO_3_ dissolution at the base of the LDE CIE (e.g. [2, 72]). In contrast to ODP Site 1209 no clear double peak for δ^13^C and δ^18^O is visible at Site 1210 and also no third peak [18].

If we attribute our benthic foraminiferal δ^18^O shift to temperature, this results in a temperature increase of ~1.6°C at the base of the LDE respectively the lowest LDE peak. Our subsurface planktic foraminiferal data would correspond to a temperature shift of ~2.4°C (0.6‰) and planktic surface data to 2.8°C (0.7‰). The non-uniform warming of the ocean could have led to water layers characterized by different temperatures, intensifying a temperature-driven upper ocean stratification.

### 4.4 Habitat implications of planktic foraminifera

The δ^13^C and δ^18^O record of benthic and planktic foraminifera display the typical inter-specific offset between benthic foraminifera showing the heaviest δ^18^O and lightest δ^13^C values, subsurface-dwellers with intermediate values for both isotope species, and surface-dwellers with the lightest values for δ^18^O and the most positive ones for δ^13^C (Figs 2 and 5). The apparent differences between surface dwelling and benthic foraminifera of nearly 2‰ for δ^13^C and 1.0 to 1.5‰ δ^18^O point towards a generally well-stratified water column as can be expected for the subtropical central Pacific. δ^13^C in photosymbiotic foraminifera tests are generally enriched in ^13^C in comparison to asymbiotic living species due to the symbionts’ preferred ^12^C consumption (e.g. [73]). δ^18^O is somewhat more negative in photosymbiotic species than in asymbiotic subsurface dwelling ones [46] (here: 0.86‰ more negative on average, N = 70). In addition to the planktic species *M*. *angulata* and *P*. *pseudobulloides/variospira*, which represent surface and subsurface habitats, several other species were analyzed to better characterize their depth habitat and to better interpret general community changes (Figs 4 and 7). Data of *M*. *velascoensis* imply a preference for the same environmental conditions as *M*. *angulata* and *M*. *praeangulata* as they are within the same isotope range. *Igorina* tends to have more positive δ^18^O values than *Morozovella*, hinting towards an occurrence during slightly cooler seasons or deeper waters than the latter one [46]. The signal might be influenced by a smaller average test size within the used grain size range and therefore less symbionts compared to the bigger *Morozovella* test, causing a more positive δ^18^O (e.g. [74, 75]. The lower δ^13^C values for *I*. *albeari* might also suggest a less intense photosymbiotic activity as proposed for other igorinids, e.g. *I*. *broedermanni* [73, 75, 76].

Due to low abundance only few specimens were measured for *Globanomalina imitata* and *Acarinina strabocella*. *Globanomalina imitata*, analyzed in two samples above the LDE shows values of δ^13^C (c. 1.8‰) and δ^18^O (c. -0.1‰) within the range of subsurface dwelling *P*. *pseudobulloides/variospira*. Therefore, a subsurface habitat is assumed for *G*. *imitata*. *Globanomalina chapmani* and *G*. cf. *pseudomenardii* show lower δ^18^O (c. -1.2‰), which is interpreted as higher habitat temperatures than *P*. *pseudobulloides*. *Acarinina strabocella* shows more ambiguous results: The δ^13^C signal of all four measurement points are closely spaced within 0.5‰ (2–2.5‰), yet the δ^18^O signal strongly varies (0–1.7‰). Therefore two samples suggest a surface ocean, photosymbiont-bearing habitat as proposed from other studies [73], whereas the two other samples suggest a non-photosymbiont thermocline habitat.

### 4.5 Planktic foraminiferal assemblages

*Praemurica uncinata*, *P*. *inconstans* and *P*. *praecursoria* show similar patterns in abundance concerning their disappearance at the lowest LDE peak (Fig 7). This suggests that this group is seriously affected by LDE-related environmental changes like carbon cycle perturbance and temperature and was less competitive compared to *Morozovella* species inhabiting a similar ecological niche. The disappearance of *Praemurica* close to this stratigraphic level has previously also been documented in shelf successions from the Tethys Ocean (e.g. [77]). A similar faunal shift has been documented for the latest Danian in the northeastern Atlantic [78], however, the LDE itself has not been identified in these successions.

The LDE at Site 1210 marks a permanent decrease in *M*. *praeangulata* and a simultaneous increase in *M*. *angulata* (Fig 7), suggesting better ecological adaptation to LDE and post-LDE surface conditions for *M*. *angulata* compared to its precursor. Above the LDE, surface water conditions favored the prevalence of *Morozovella*. Most *Morozovella* species show a long-term trend to higher relative abundances and seem to be more adapted to warm and stratified oligotrophic surface water.

Thermocline dwelling *P*. *pseudobulloides/variospira* was a highly successful species during the LDE as highest relative and absolute abundance were found between the two LDE peaks suggesting that *Parasubbotina* benefited from oceanic changes accompanying the LDE (Fig 7). The rise to higher abundances starts together with the onset of the negative CIE which again hints towards an environment enriched in nutrients in form of digestible particles as preferred by subsurface dwellers. However, directly above the LDE *Parasubbotina* was again less successful in exploiting a niche in the thermocline. The closely related *Subbotina* spp. is rather rare in the late Danian which does not allow for a detailed ecological interpretation.

A low-resolution study in the Tethys Ocean [77] at three shelf locations in Tunisia over the P2-P3b time period comprises slightly different results from ours. There, *Praemurica* spp., *Subbotina* spp. and *Parasubbotina* spp. are slightly more or equally abundant than *Morozovella* spp. In Tunisia, *Subbotina* spp. was notably more abundant than *Parasubbotina* spp. [77]. This is not the case in our study, where *Morozovella* is by far most abundant, followed by *Parasubbotina*, *Igorina*, *Subbotina* and *Praemurica*. However, these differences in assemblage composition might be largely attributed to the fact that these localities are situated on the North African continental margin at much shallower paleodepths (outer neritic to upper bathyal).

**Fig 8:**
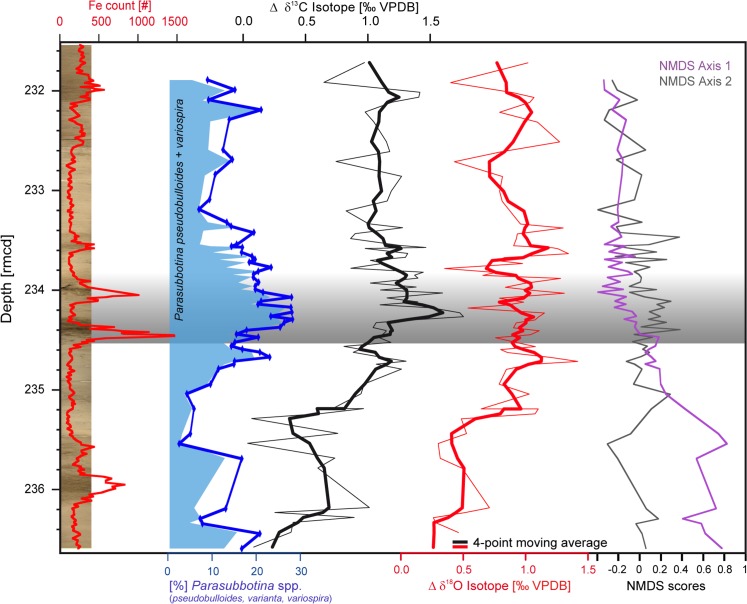
δ^13^C and δ^18^O gradient and faunal development. Gradient of δ^13^C and δ^18^O signals calculated by planktic surface minus subsurface dwelling foraminifera, expressed as Δδ^18^O and Δδ^13^C. A four-point moving average has been applied. The core photo and the XRF core scanning Fe counts serve for stratigraphic correlation. In addition results from non-metric multidimensional scaling (NMDS) as a statistical measure of the faunal composition are shown (see also Fig 9). The LDE is marked in grey. Data available in S1 and S2 Tables.

**Fig 9:**
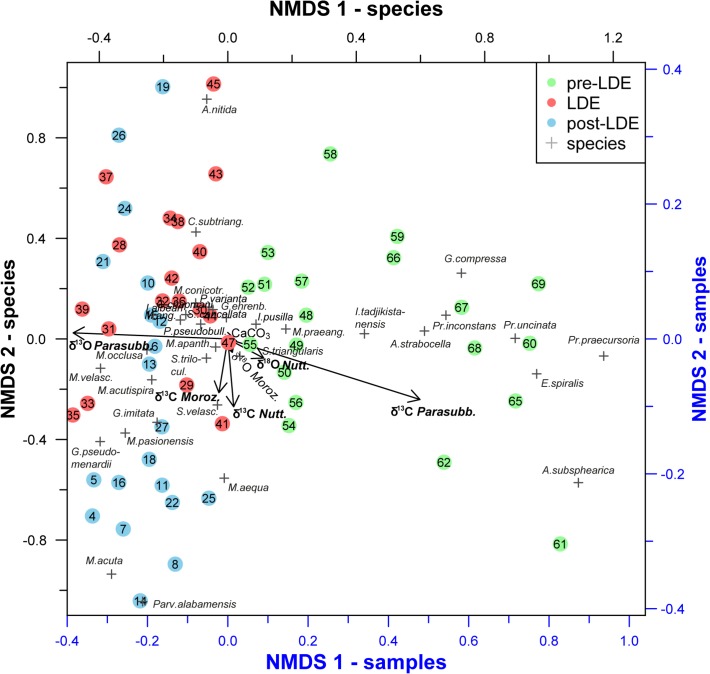
Non-Metric multidimensional Scaling. Results of multivariate analysis (non-metric multidimensional scaling, NMDS, stress = 0.17) showing differences of faunal communities (species-level) between the event stages. Most obvious is a strict division between pre-LDE (green, >234.45 rmcd, numbers 48–59) and post-LDE fauna (blue, <233.75 rmcd, numbers 1–27), whereas the event fauna (red, numbers 28–47) is positioned between the previous two phases, in a mixed position within post-event samples. Numbers refer to samples numerated from top to bottom (cf. S1 Table). From the fitted environmental vectors, the δ^13^C and δ^18^O of subsurface dwelling Parasubbotinids are related to the major NMDS axis controlling the distribution. Only the vectors of δ^13^C and δ^18^O of the parasubbotinids are significant at the 95% level.

NMDS ordination (Figs 8 and 9) reveals a development of our paleocommunity (Fig 7) during the investigated interval by projecting the ranking of relationships between species and samples as well as environmental proxies like δ^13^C and δ^18^O into two dimensions. A distance of the samples between before, during and after the event is apparent, specifically along NMDS axis 1 (Fig 8). The fauna during and above the LDE shows a close connection, whereas pre-LDE samples differ from the later faunal communities, seen by scores (Fig 8) as well as distances in the scatter plot (Fig 9). Post-LDE samples appear to be relatively homogeneous in their assemblages and reflected by the constantly very low NMDS axis 1 scores after the LDE. Subsurface δ^13^C and δ^18^O (*P*. *pseudobulloides/variospira*) strongly correspond to NMDS axis 1 (Fig 8) but even more striking are similarities to the δ^13^C and δ^18^O offset gradient as discussed in higher detail below.

### 4.6 Paleoceanographic implications

Non-photosymbiotic species show in general a higher variability in δ^13^C and δ^18^O values than photosymbiotic surface dwellers, most likely due to their light-independent ability to float in vertical motion, which is useful for environmental adaptation in aspects of nutrition and habitat temperature [73, 79]. *Parasubbotina* has been interpreted as a subsurface thermocline dweller [46, 47], which is well supported by our δ^13^C and δ^18^O data of *P*. *pseudobulloides/variospira*. As discussed above, during the ~200 kyr of the LDE, thermocline dwellers (*P*. *pseudobulloides/variospira*, *G*. *chapmani* and G. *imitata*) rise in abundance and decline above the event. It is likely that these species have followed a nutricline or temperature change with optimal living conditions and seem to have benefited from a more stratified upper water column during the LDE. A potential scenario explaining the abundance changes of *Parasubbotina* might be a thermocline shallowing and an accompanied development of a deep chlorophyll maximum (DCM). A DCM is a feature of highly stratified open oceans and is often either seasonally formed or part of a permanent gyre system [80, 81] when the thermocline, or more generally speaking the pycnocline, is positioned well within the euphotic zone. In such a setting the surface mixed layer is usually characterized by oligotrophic conditions, and nutrients are trapped right below the pycnocline. This allows for the development of a DCM between the pycnocline and the base of the euphotic zone. Due to consumption of nutrients by the deep-phytoplankton community the nutricline lies at the base of the DCM [82]. A comparable scenario has been proposed for the formation of Mediterranean Sea sapropels during the Late Quaternary. Here, enhanced stratification occurs during warm phases due to salinity and temperature changes in favor of stagnation and low oxygen conditions at the sea floor [83, 84]. However, other studies attribute the lack of vertical mixing and deep water ventilation in the Mediterranean Sea to increased density stratification caused by freshwater inflow (e.g. [85]).

Directly above the second LDE peak light-dependant surface dwellers (*I*. *albeari*, *M*. *angulata* and *M*. *conicotruncata*) show higher abundances while thermocline layer inhabitants decline, probably resulting from a return to conditions similar to the pre-event stratified upper ocean.

At Shatsky Rise unequal warming of the different water masses during the LDE, with stronger warming of the surface mixed layer may have enhanced stratification of the upper water masses. Both δ^13^C and δ^18^O data suggest a stronger surface to subsurface gradient from slightly below the LDE upwards (Fig 8). The δ^13^C gradient is more pronounced than the δ^18^O one and parallels the relative abundance of the subsurface-dweller *Parasubbotina*. The difference in the offset between before and after the LDE makes c. 0.8‰ for δ^13^C and c. 0.6‰ (≙ c. 2.4 °C) for δ^18^O on average. Both δ^13^C and δ^18^O gradients are rising by 0.5‰ 20 cm below the LDE and remain higher after the event than before. Above the LDE at 233.7 rmcd a slight reduction of these gradients took place. The δ^13^C gradient is largely controlled by the signature of *P*. *pseudobulloides/variospira* whereas surface dwelling foraminifera show more stable values (Figs 2 and 5). The δ^18^O offset gradient is more balanced and not controlled by one habitat alone, but might be interpreted as a stronger temperature increase of the surface ocean whereas thermocline waters are influenced by somewhat cooler and/or more saline waters.

Due to the co-variation between the δ^13^C isotope gradient and the relative abundance pattern of *P*. *pseudobulloides/variospira* as well as the importance of the *Parasubbotina* δ^13^C in the NDMS we presume that *P*. *pseudobulloides/variospira* took advantage of the well stratified ocean accompanied by the formation of a prominent DCM, and, thus, high nutrient availability in subsurface waters as well as less competitive pressure due to the disappearance of aforementioned species.

Alternatively, the long-term trend of an increasing gradient strength with a weaker gradient below and the observed variability above the LDE might be also partly owing to the Paleocene paleogeographic position of Shatsky Rise on the northern rim of the equatorial divergence zone (Fig 3). Intervals with a weaker gradient might indicate periods of enhanced upwelling. Since the gradient did not fully recover to pre-LDE levels in our record, we, further, tentatively speculate that Shatsky Rise passed a certain threshold inhibiting or reducing vertical mixing by leaving the equatorial upwelling zone due to its plate tectonic movement [38].

Finally, the timing of the faunal change in relation to the LDE is crucial for understanding the planktic foraminiferal faunal response to the LDE. From the isotope depth gradients and δ^18^O data as well as the NMDS axis 1 scores it is apparent that substantial changes in the fauna, but also in the oceanographic conditions commenced ~170 ky before the LDE onset (~0.7 m below, Figs 2 and 8). However, a second step in these changes corresponds to the short-term negative δ^13^C excursion during the LDE. Our data indicate long-term ocean warming and, thus, stratification started well before the LDE, but culminated during the LDE interval. This let us conclude that both long-term and transient environmental changes associated with the LDE had a strong control on the assemblage composition of planktic foraminifera (Fig 7).

## Conclusions

High-resolution analysis of planktic foraminiferal assemblages and foraminiferal δ^13^C and δ^18^O provide new insights into the prevailing paleoceanographic conditions and planktic foraminiferal responses to the Latest Danian Event at Shatsky Rise:

1. Some dissolution is apparent from the studied samples of the LDE at ODP Site 1210 (Shatsky Rise) during the LDE Fe peaks, and also recrystallization might be an issue in some intervals of the carbonate-rich intervals before and after the LDE. Eight samples were excluded from the faunal analysis for this reason.
2. A significant negative CIE of 0.7‰ in surface, 0.9‰ in subsurface dwellers and 0.6‰ in benthic foraminifera was observed, identifying the LDE together with the two prominent Fe peaks.
3. The entire water column has been warmed by ~1.6–2.8°C (0.4‰ benthic, 0.6‰ subsurface and 0.7‰ surface dwellers) during the LDE at Shatsky Rise, but started ~170 ky below the LDE onset. This decrease in δ^18^O supports the idea that this event might represent a Paleocene hyperthermal, albeit temperature variability is generally of a similar magnitude throughout the latest Danian. However, this is the only warming phase that is accompanied by a negative CIE in the mid-Paleocene.
4. The isotope gradients between surface and subsurface dwellers point towards an enhanced stratification of the upper water column just below the LDE, strongly enhanced during it and less but still enhanced above. This change in the gradient is accompanied by abundance changes of the subsurface dwelling *Parasubbotina pseudobulloides/variospira* probably linked to the development of a deep-chlorophyll maximum under well-stratified conditions. Above the LDE, symbiont-bearing surface dwellers (*Morozovella*) become more successful after they might have developed more effective photosymbiosis strategies.
5. We observed major changes in the faunal assemblages specifically in photosymbiont-bearing taxa like the abrupt virtual disappearance of *Praemurica* within the first LDE peak and the gradual evolution of new *Morozovella* taxa.
6. Multivariate statistics indicate rather distinct faunal communities below, during and above the event. The results clearly show that faunal changes started more than ~170 kyr before the LDE similar to the isotope depth gradients and temperature changes.
7. The first appearance datum (FAD) of *Igorina albeari* is dated well below (> 400 kyr) the onset of the LDE, and not at the LDE (or directly below) as proposed in other studies.

## Supporting Information

S1 Table Samples. List of used samples for this work comprising also the excluded ones due to bad preservation. Further a list of NMDS scores (Figs 8 and 9) with sample ID.
(XLSX)

S2 Table Isotopes Planktic Foraminifera. All measured isotope data of planktic foraminifera as used in Figs 2, 5 and 8.
(XLSX)

S3 Table Isotopes Benthic Foraminifera. All measured isotope data of benthic foraminifera as used in Figs 2 and 5.
(XLSX)

S4 Table Parameters. Carbonate content, fragmentation, coarse fraction, planktic foraminifera proportion, planktic foraminifera number, planktic foraminifera accumulation rate, sedimentation rate and the two diversity indexes simple diversity and Shannon H’. Data are used in Fig 4.
(XLSX)

S5 Table Planktic foraminifera count data. Simple planktic foraminifera count data as used in Figs 7 and 9 with fraction size.
(XLSX)
